# VB-84922 is a small molecule that inhibits ER-to-golgi transport of SREBPs-SCAP complexes

**DOI:** 10.3389/fphar.2026.1732319

**Published:** 2026-03-24

**Authors:** J. Jose Corbalan, Wiebke Schormann, Pranavi Jagadeesan, Justin Kale, Yu-Chiang Huang, Rachael Siegel, James R. Beasley, Jyun-Peng Tung, Axel Nohturfft, David Andrews, Joseph T. Nickels

**Affiliations:** 1 The Institute of Metabolic Disorders, Genesis Research and Development Institute, Genesis Biotechnology Group, Hamilton, NJ, United States; 2 Sunnybrook Research Institute, Toronto, ON, Canada; 3 Venenum Biodesign Inc., Genesis Biotechnology Group, Hamilton, NJ, United States; 4 City St. George’s, University of London, London, United Kingdom; 5 Rutgers Center for Lipid Research, New Jersey Institute for Food, Nutrition, and Health, Rutgers University, New Brunswick, NJ, United States

**Keywords:** cholesterol, fatty acid, inhibitor, INSIG, lipid, SCAP, SREBP, transcription

## Abstract

The sterol response element binding proteins (SREBPs), SREBP-1a/c and SREBP-2, are sterol-regulated transcription factors that control the expression of cholesterol and fatty acid-raising genes. Elevated expression of SREBPs has been linked to increased morbidity and mortality rates associated with conditions including obesity, cancer, and cardiovascular disease. Therefore, the development of new therapeutics to inhibit SREBP activity may be beneficial for treating various diseases associated with altered lipid levels. In their inactive state, SREBPs remain sequestered in the ER membrane in a complex with SREBP cleavage activating protein (SCAP) and one of two ER-anchoring proteins, Insig-1 or Insig-2. Activation proceeds through dissociation of SREBP/SCAP from Insigs, SCAP-assisted translocation to the Golgi, proteolytic membrane release and nuclear import. We employed a high-throughput enzyme complementation assay to identify inhibitors of SREBP-2 translocation to the nucleus, resulting in the identification of VB-84922 having an IC_50_ value of 0.45 ± 0.052 μM. VB-84922-mediated inhibition of nuclear translocation was confirmed by fluorescence microscopy with an mNeonGreen-SREBP-2 fusion protein. Crucially, VB-84922 inhibited the lovastatin-induced activity of an SREBP-responsive reporter construct and suppressed the expression of endogenous SREBP target genes. Co-transfection assays using an SREBP reporter and fluorescence microscopy were used to delineate the target of VB-84922 in the SREBP activation pathway. The drug blocked ER export of wild-type SCAP but had no effect on SREBP activity in cells expressing the nuclear form of SREBP-1a, or mutated versions of SCAP that are unable to bind Insigs and that chaperone SREBP to the Golgi constitutively. These results suggest that VB-84922 targets a step upstream of ER export in the SREBP activation cascade. VB-87496, a therapeutic lead compound, developed from VB-84922, demonstrated *in vivo* efficacy within a murine acute fasting-refeeding model by inhibiting full-length SREBP protein maturation and SREBP-dependent transcription. VB-87496 represents a specific SREBPs-SCAP inhibitor that has potential for further lead optimization medicinal chemistry efforts to generate a potent and selective pre-clinical candidate for treating lipid-related diseases.

## 1 Introduction

Hyperlipidemia is a widespread health concern contributing to high healthcare costs (Ferrara et al., 2021). Mixed hyperlipidemia, marked by increased triglycerides and cholesterol, increases the risk of cardiovascular disease, obesity, and stroke (Ferrara et al., 2021). Statins like Lipitor and rosuvastatin effectively lower LDL cholesterol but can cause side effects such as gastrointestinal distress, joint pain, and, rarely, rhabdomyolysis (Azemawah et al., 2019; Cai et al., 2021). Resistance to statins may occur, making monotherapy insufficient for optimal LDL-C reduction (Danilov et al., 2024). PCSK9 (proprotein convertase subtilisin/kexin) inhibitors - PCSK9 is a proteolytic enzyme that increases serum LDL-cholesterol (LDL-C) through degrading the LDL receptor (Danilov et al., 2024)- are generally well-tolerated but may also cause gastrointestinal issues and cognitive side effects (Roth and Davidson, 2018; Gurgoze et al., 2019). Other therapeutics include Bempedoic acid, an ATP citrate lyase inhibitor that is approved for treating patients with heterozygous familial hypercholesterolemia (HeFH) and atherosclerotic cardiovascular disease (ASCVD) (Paton, 2020), as well as fenofibrates such as Tricor (Rosenson, 2008) and omega-3 fatty acids for lowering triglycerides (Zhang et al., 2025). As the American Heart Association and the American College of Cardiology continue to recommend lower optimal LDL-cholesterol levels for individuals, there is ongoing interest in identifying additional therapeutic options for treating hyperlipidemia (Arnett et al., 2019; Gautam et al., 2025).

The SREBP-1a/c and SREBP-2 proteins function as endoplasmic reticulum (ER)-tethered transcription factors that regulate *de novo* synthesis of fatty acids and cholesterol (Brown and Goldstein, 1997; 1999). Under conditions of elevated cholesterol, these proteins remain anchored to the ER, associated with SCAP (Eberle et al., 2004; Brown et al., 2018), which in turn interacts with insulin signaling proteins 1 and 2 (INSIGs) (Rawson, 2003), which share 59% homology (Yabe et al., 2002). The association of SCAP with either INSIG1 or INSIG2 is mediated by direct binding of cholesterol to the sterol-sensing domain of SCAP, thereby stabilizing INSIG1/2-SCAP complexes, which inhibits translocation of SREBPs-SCAP complexes to the Golgi apparatus when cholesterol levels are high (Yang et al., 2002; Williams et al., 2026). Conversely, when cholesterol levels are reduced, SCAP undergoes a conformational change leading to dissociation from INSIGs, thus permitting SREBPs-SCAP trafficking to the Golgi by a COPII-mediated process (Yang et al., 2002; Yan et al., 2021). In the Golgi, SREBPs are sequentially cleaved by Site-1 (S1P) (Duncan et al., 1997) and Site-2 (S2P) proteases (Duncan et al., 1998), resulting in the release of soluble amino-terminal transcription factors that translocate to the nucleus initiating fatty acid and sterol gene transcription.

It is well-established that SREBP transcription must be strictly regulated, as changes can disrupt lipid balance and cause disease. Mice overexpressing SREBP-1a have increased triglyceride and cholesterol levels in the liver (Shimano et al., 1996; Horton et al., 1998), while liver-specific SCAP deletion impairs *de novo* fatty acid and cholesterol biosynthesis and results in lower hepatic cholesterol and triglyceride levels (Matsuda et al., 2001). Mice expressing the SCAP^D443N^ mutant are resistant to cholesterol feedback inhibition and show higher expression of cholesterol-related genes and elevated liver cholesterol and triglycerides (Korn et al., 1998). Conversely, overexpression of *INSIG1* in mouse liver reduces insulin-stimulated, SREBP-1c-dependent *de novo* lipogenesis (Engelking et al., 2004). In humans, increased expression of *SREBF1* and *SREBF2* has been reported in association with obesity, cardiovascular disease, and cancer, which may be related to the morbidity and mortality observed in these conditions (Kolehmainen et al., 2001; Xue et al., 2020; Zhou et al., 2022; He et al., 2024).

Small molecules that inhibit SREBP translocation, potentially by binding to and decreasing SCAP activity—fatostatin, lycorine, betulin, and dipyridamole—have demonstrated *in vivo* effectiveness in mouse models. *ob*/*ob* mice on a high fat diet and treated with fatostatin have reduced body weight gain, fat accumulation, and hyperglycemia (Kamisuki et al., 2009), while administering lycorine, which stimulates SCAP degradation, lowers hyperlipidemia, insulin resistance, and hepatic steatosis (Zheng et al., 2021). Western diet-fed mice given betulin show improved hyperlipidemia and insulin sensitivity (Tang et al., 2011). Mice given high-dose dipyridamole show lower levels of mature forms of SREBP-1a/c and SREBP-2 in an acute fasting/refeeding study (Esquejo et al., 2021). Therefore, blocking SREBPs-SCAP translocation represents a potentially effective strategy for addressing diseases related to aberrant lipid metabolism.

VB-84922 has been identified as an ER to Golgi translocation inhibitor of SREBPs-SCAP complexes. Administration of VB-84922 to cells retains SREBP-2-SCAP in the ER under low cholesterol-inducing conditions, leading to decreased *SRE*-dependent transcription and reduced SREBP-2 nuclear accumulation. Additionally, HepG2 cells treated with VB-84922 exhibit lower SREBPs-dependent gene expression and mature processed SREBP-1c and SREBP-2 proteins. VB-87496, a chemically optimized derivative of VB-84922, demonstrates *in vivo* efficacy in inhibiting SREBP maturation and SREBP-dependent gene transcription.

## 2 Materials and methods

### 2.1 Miscellaneous reagents

We obtained DMEM/F12 plus Glutamax medium (Gibco, Grand Island, NY) and oligonucleotides from Life Technologies (Paisley, United Kingdom); fetal bovine lipoprotein-deficient serum (AlphaDiagnostics, San Antonio, TX), lovastatin (mevinolin; Apexbio, Houston, TX); PF-429242 dihydrochloride (Aobious, Gloucester, MA); and 25-hydroxycholesterol (Cayman, Ann Arbor, MI) from Cambridge Bioscience (Cambridge, United Kingdom); DL-mevalonolactone (M4667), fetal bovine serum (F9665) and penicillin/streptomycin from Merck Life Science (SigmaPaisley, United Kingdom). Mevalonic acid solution was prepared from mevalonolactone as described (Bird et al., 2025).

### 2.2 Plasmids

Plasmid phRL-CMV (expressing *Renilla reniformis* luciferase) was purchased from Promega (Madison, WI) and pcDNA3 was from Invitrogen (Carlsbad, CA). Plasmids pTK-SCAP, pTK-SCAP (D443N) and pTK-SCAP (Y298C) (Nohturfft et al., 1998a), pTK-HSV-SREBP-2 (Hua et al., 1996), pCMV-INSIG1-Myc (Yang et al., 2002) and pLDLR-Luc2P (expressing destabilised firefly luciferase from the human LDL receptor promoter) have been described (Bird et al., 2025). pLDLR-Luc2P contains a DNA sequence encompassing nucleotides −481 to −91 of the 5′UTR of the *LDLR* gene. The SRE binding site within the promoter is ATCACCCCAC (Wang et al., 1993).

### 2.3 Cell culture

Medium A refers to DMEM/F12 supplemented with 10% fetal bovine serum (FBS) and antibiotics (100 units/mL penicillin and 100 mg/mL streptomycin sulfate). Medium B refers to DMEM/F12 supplemented with 5% lipoprotein-deficient serum (LPDS), 50 µM mevalonate, and 0.5 µM lovastatin plus antibiotics. CHO-K1 cells (ATCC CCL-61) were purchased from LGC Ltd. (Teddington, United Kingdom) and maintained in medium A at 37 °C in an atmosphere containing 5% CO_2_. All cell media reagents were obtained from Millipore Sigma (Rockville, MD).

### 2.4 Plasmid transfection and enzyme assays

CHO-K1 cells were transfected using TurboFect™ reagent according to the manufacturer’s instructions (Thermo Fisher Scientific, Wortham, MA) at a ratio of 3 µL Turbofect per µg DNA. To measure the activities of firefly and *R. reniformis* luciferases, cells in 96-well plates were lysed and analyzed with Dual-Glo Luciferase Assay reagents (Promega, Madison, WI) as described (Zhang and Nohturfft, 2008).

### 2.5 PathHunter high throughput assay

U2OS cells were cultured in McCoy’s 5a modified medium containing 10% FBS (ATCC #30-2007) until they reached 70% confluency. For the assay, 4 µL of 20 μM U-18666A were added to each well (1536-well format) and dried down at 37 °C for 6–8 h. Plates could be stored at room temperature at this point until use. Compounds to be tested were resuspended in DMSO and an 11 point concentration range was used for testing (0.0017 μM–100 μM). 40 nL of compound was added in duplicate to assay plates using an Acoustic dispenser. U2OS cells (∼5,000 cells) were resuspended in 8 μL of DiscoverRx (Fremont, CA) assay complete cell plating reagent and added to each well. Plates were briefly centrifuged and then grown for 24 h at 37 °C and 5% CO_2_. After 24 h, 4 μL of Pathfinder detection reagent was added to each well. Plates were briefly centrifuged, incubated for 60 min in the dark and read on an PerkinElmer EnVision Microplate Reader.

### 2.6 *LDLR*-*SRE*-luciferase screening assay

HepG2 cells were maintained in minimal essential medium (MEM) supplemented with 10% FBS, 100 IU/mL penicillin, 100 μg/mL streptomycin, 2 mM Glutamax, non-essential amino acids, and 1 mM sodium pyruvate at 37 °C and 5% CO_2_. Approximately 10,000 cells in 20 μL were dispensed into 96-well plates containing different concentrations of the test compound and incubated for 48 h at 37 °C and 5% CO_2_. Cells were lysed using One Glo™ lysis buffer (Promega, (Madison, WI), followed by centrifugation and mixing on a plate shaker for 40 s. Plates were then equilibrated for 30 min at room temperature. Subsequently, 25 μL of One Glo™ luciferase reagent was added to each well, and luminescence was measured after 3 min using a PerkinElmer EnVision Microplate Reader. The SRE-Luc plasmid contains nucleotides −481 to −91 of the *LDLR* 5′UTR with the SRE site, ATCACCCCAC.

### 2.7 β-lactamase enzyme assay

Recombinant β-lactamase (AmpC) was sourced from Abcam (Waltham, MA) and prepared at 13 μg/mL (0.42 μM) in 50 mM phosphate buffer (pH 8.0). For the assay, 2.5 μL of AmpC stock (0.014 μM) was added to 75 μL buffer A with 0.02% Triton X-100 in 384-well plates. After centrifugation (1,000 rpm, 1 min) and a 5-min equilibration, 1.5 μL nitrocefin was added and mixed. Samples were incubated in the dark for 2 h before measurement on a PerkinElmer EnVision plate reader. Tazobactam (Millipore Sigma, Burlington, MA) was used as a positive inhibition control.

### 2.8 Cell lines and culture conditions for high content screening

NMuMG cells were cultured in DMEM, containing 10 μg/mL bovine insulin (Millipore Sigma, (Rockville, MD), 10% FBS (Gibco, Grand Island, NY), and penicillin/streptomycin (Wisent, QC, Canada). The packaging cell line HEK293T were grown in DMEM (Gibco), supplemented with 10% FBS and penicillin/streptomycin. All cell lines were maintained in a 5% CO_2_ atmosphere at 37 °C.

### 2.9 Plasmid construction and stable cell line generation

The mNeonGreen-SREBP-2 fusion protein was constructed by using the PCR-amplified coding region of the SREBP-2 gene and the lentiviral vector pLVX-EF1a-mNeonGreen-IRES-Neomycin by employing the restrictions sites NotI and SpeI (New England BioLabs, Ipswitch, MA). As a nuclear marker, RanGTP and other organelle markers were fused to mScarlet-I in the following lentiviral vector pLVX-EF1a-mScarlet-I-IRES-Puromycin using BamHI (NEB)and SpeI (NEB). The stable expression of fusion proteins was achieved by transduction of normal murine mammary gland (NMuMG) cells. Briefly, lentiviral DNA plasmid and pPAX2 and pMD2. G plasmids were transfected into HEK293T cells at 1:1:0.1 ratios utilizing polyethylenimine (PEI, Polysciences). After 72 h s, NMuMG cells were transduced with filtered (0.45 mm) virus-containing supernatant collected from the HEK293T cells. Stably expressing cells were selected by applying 400 μg/mL G418 (Wisent) and 2 μg/mL puromycin (Millipore Sigma, Rockville, MD).

### 2.10 SREBP-2 translocation assay

NMuMG cells expressing mNeonGreen-SREBP-2 and individual tagged organelle markers were seeded in a microplate (PhenoPlate™ 384-well microplate, Revvity, Waltham, MA) and grown for 24 h. Cells were treated with U18666A for 16 h, followed by imaging with a spinning disk automated confocal microscope (OPERA, Phenix, Revvity) with a ×40 water objective (NA 1.1) in a defined temperature (37 °C) and CO_2_ (5%) environment. Four images acquisition sCMOS cameras (4.6 MP, 16-bit) were used. Cells were imaged with mNeonGreen and mScarlet-I filter sets, using laser lines at 488 nm and 561 nm for excitation, respectively. Emission signals were acquired in the ranges of 500–550 nm for mNeonGreen and 570–630 nm for mScarlet-I. Cell segmentation and feature extraction were carried out in CellProfiler software (version 4.1.3) (McQuin et al., 2018). Subcellular localization was assigned by classifying the cell images using Random Forests algorithm as described in Schormann et al., (Schormann et al., 2020). The reference subcellular image library data was uploaded to the Image Data Resource (https://idr.openmicroscopy.org/webclient/?show=screen-2952).

### 2.11 HepG2 cell *LDLR-SRE* luciferase inhibitor assay

HepG2 cells were maintained in DMEM supplemented with 10% FBS, 100 IU/mL penicillin, and 100 μg/mL streptomycin. Cells were co-transfected with plasmids phRL-CMV and pLDLR-Luc2P (Bird et al.). Cells were transferred to 96-well plates and cultured in LPDS medium supplemented with 10 mM lovastatin for 16 h to induce *LDLR-SRE* luciferase promoter activity, either in the absence or presence of the specified drug concentrations. Cells were lysed with One GloTM lysis buffer (Promega, Madison, WS), centrifuged, and plates equilibrated for 30 min at room temperature. Then, 25 μL One GloTM luciferase reagent was added per well, and luminescence was measured after 3 min using a PerkinElmer EnVision Microplate Reader.

### 2.12 Protein extraction

HepG2 cells were lysed in RPMI buffer (Millipore Sigma, Rockville, MD. Cell lysates were obtained through low-speed centrifugation and kept at −20 °C until use. Protein concentrations were measured using the Pierce™ BCA Protein Assay Kit (Waltham, MA).

### 2.13 Western analysis

Proteins were separated by SDS-PAGE, transferred to nitrocellulose membranes, and blocked with 10% milk in TBST. Membranes were incubated with primary antibodies (12–16 h) and secondary antibodies (1–4 h), with several washes in between, then visualized using a chemiluminescent reagent and Amersham Imager 600; GAPDH served as a loading control. Each western blot panel derived from a single gel, with blots stripped and reprobed for different targets as needed. Densitometry was performed after adjusting TIFF image brightness to 150 in Photoshop. Antibodies are listed in Supplementary Table S1.

### 2.14 qRT-PCR assay

Total RNA was isolated from cells with the RNeasy Plus Universal kit (Qiagen, Germantown, MD) and treated with RNase-free DNase. Reverse transcription was performed using the QuantiTect kit (Qiagen, Germantown, MD), followed by PCR amplification with the Power SYBR RNA-to-CT 1-step kit (ThermoFisher, Bridgewater, New Jersey).

### 2.15 Statistical analysis

Statistical analysis was performed using two-way ANOVA with Tukey’s *post hoc* in GraphPad V10.6.1. Results are presented as mean ± SD.

### 2.16 *In vivo* fasting-refeeding study

Figure 9 provides an overview of the study cohorts and design. Male C57BL/6 mice, approximately 6 weeks old, were acclimated to the facility prior to the study. The animals subsequently underwent adaptation to an altered light/dark cycle so that feeding would occur during the dark phase, coinciding with their natural feeding behavior. Following acclimation, the mice were fasted for 24 h. Select groups received either 120 mg/kg dipyridamole (PEG/tartaric acid; ip) or 50 mg/kg VB-87496 (0.5% Methocel; PO) 1 hour before the end of the fasting period. Designated groups were then refed chow (Research Diets #D13221) for 7 h, after which they were euthanized and liver tissue was collected for analysis. (Cheng et al., 2022). The Invivotek institutional animal care and use committee approved all animal studies. All studies followed procedures according to the “*Institutional Animal Care and Use Committee Handbook*”.

## 3 Results

### 3.1 VB-84922 inhibits SREBP-2 nuclear translocation and sterol response element-dependent promoter activity

The PathHunter eXpress SREBP-2 Nuclear Translocation Assay was used to screen the 5.5 million compound ECLiPs library (Diller and Hobbs, 2004) for inhibitors of SREBP-2 translocation. The assay employs enzyme fragment complementation technology with an ER-tethered SREBP-2 tagged with a donor fragment (ED) and an enzyme acceptor fragment (EA) tethered to the nucleus (Supplementary Figure S1). Upon activation, a soluble SREBP-2-ED tagged protein translocates to the nucleus, where it interacts with the nuclear EA fragment, generating a measurable signal.

U2OS human osteosarcoma cells were treated with the intracellular cholesterol transport inhibitor U18666A to induce translocation and compounds that reduced the activated signal were identified. U18666A binds to the sterol-sensing domain of the Niemann-Pick C1 protein and blocks lysosomal cholesterol export generating a cholesterol deplete-like condition (Underwood et al., 1996; Lu et al., 2015).

Initial screening identified nine sub-libraries, and resynthesis of individual templates from each inhibited SREBP-2 translocation with IC_50_ values ranging from 0.44 ± 0.072 µM to 2 ± 0.15 µM. Based on the chemical structures and predicted medicinal chemistry feasibility based on sub-library pharmacophores, VB-84922 was selected for further characterization.

**FIGURE 1:**
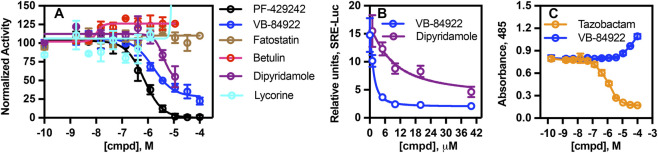
VB-84922 inhibits SREBP-2 nuclear translocation. **(A)** U2OS cells were treated with 10 μM U18666A for 16 h in the absence or presence of the indicated concentrations of each drug and SREBP-2 nuclear translocation was determined using a PerkinElmer EnVision microplate reader. **(B)** HepG2 cells harboring an *LDLR-SRE*-luciferase reporter plasmid were treated with 10 μM lovastatin and the indicated concentrations of dipyridamole or VB-84922 for 16 h. Luciferase activity was determined using a PerkinElmer EnVision microplate reader. **(C)** β-lactamase activity was determined in the absence or presence of the indicated concentrations of Tazobactam or VB-84922 using a PerkinElmer EnVision microplate reader.

VB-84922 demonstrated an IC_50_ value of 0.45 ± 0.052 μM in the PathHunter assay (Figure 1A, open blue circles), inhibited *LDLR*-*SRE*-luciferase promoter activity expressed in HepG2 cells with an IC_50_ value of 2.3 ± 0.21 µM (Figure 1B, open blue circles) and did not exhibit non-specific inhibition of β-lactamase activity (Figure 1C, open blue circles). The *LDLR*-*SRE*-luciferase construct drives promoter activity from a fragment of the *LDL* receptor promoter that contains a high-affinity SREBP binding site (*SRE*) (Briggs et al., 1993; Wang et al., 1993).

Increasing concentrations of dipyridamole also resulted in a reduction in translocation signal (Figure 1A, open purple circles), with a calculated IC_50_ value of 3.9 ± 0.43 μM and inhibited promoter activity (IC_50_ = 12.8 ± 1.2 μM) (Figure 1B, purple circles). VB-84922 showed stronger inhibition than dipyridamole, reducing promoter activity to 10% of control levels resulting in an average 2.3-fold decrease in total area under the curve based on analyses of n = 6 assay replicates (300.2 + 9.7 vs. 124.6 ± 11.1; *p ≤ 0.0001*). Fatostatin (Figure 1A, open brown circles), betulin (Figure 1A, open red circles), and lycorine did not inhibit activity in either the translocation (Figure 1A, open cyan circles) or promoter assays (not shown).

### 3.2 VB-84922 reduces SREBP-1c and SREBP-2 proteolytic processing in HepG2 liver cells

Blocking SREBPs-SCAP translocation is expected to lower mature SREBP-1a/c and SREBP-2 levels, suppressing their transcriptional activity. To test if reduced translocation led to decreased activity, human HepG2 cells were treated with lovastatin and either 25-HC or different doses of VB-84922 for 16 h, then *SREBF1* and *SREBF2* mRNA, as well as full-length and processed SREBP-1c and SREBP-2 proteins, were measured.

Studies using human cell cultures were essential to confirm whether VB-84922 is effective, since the drug must work in treating human lipid disorders. If it failed to show efficacy at this stage, VB-84922 would not be considered a promising candidate for further medicinal chemistry development.

**FIGURE 2:**
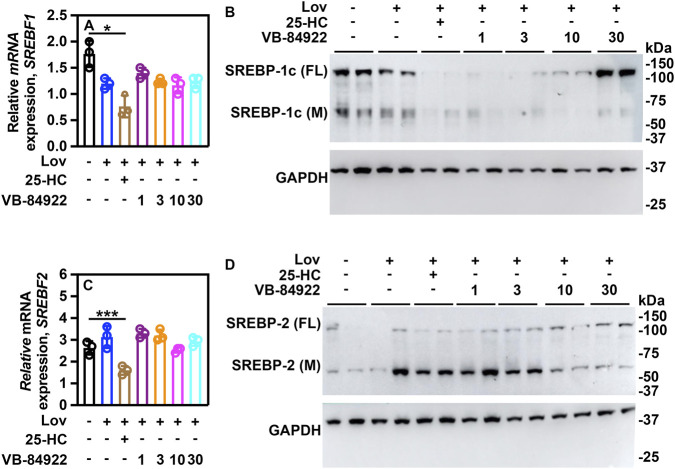
VB-84922 inhibits the proteolytic processing of SREBP-1c and SREBP-2. HepG2 cells were incubated with or without 10 μM lovastatin for 16 h and the indicated concentrations of VB-84922. **(A)** relative mRNA expression of *SREBF1*. **(B)** protein levels of full-length and mature forms of SREBP-1c **(C)** relative mRNA expression of *SREBF2*. **(D)** protein levels of full-length (FL) and mature (M) forms of SREBP-2). A two-way ANOVA (Tukey’s *post hoc*) was used for statistical analysis. Data are shown as mean ± SD. *p < 0.01; ***p < 0.0001.

Expression of *SREBF1* and *SREBF2* mRNA was observed in HepG2 cells treated with LPDS containing 10 mM lovastatin (Figures 2A,C). Administration of 25-HC led to a decrease in *SREBF1* and *SREBF2* expression in lovastatin-treated cells (Figures 2A,C). Gene expression levels remained unchanged with VB-84922 treatment. These findings are consistent with earlier research conducted in comparable cell culture systems (Scharnagl et al., 2001; Dubuc et al., 2004; Jeong et al., 2008).

Western blot analysis showed full-length (FL) and mature (M) SREBP-1c in lovastatin-treated cells (Figure 6B). Adding 2.5 μM 25-HC completely eliminated mature SREBP-1c, and all doses of VB-84922 also caused its loss.

Low levels of full-length SREBP-2 were observed under all treatments (Figure 2D). Mature SREBP-2 was depleted in lovastatin-treated cells exposed to 10 and 30 μM VB-84922 (Figure 2D).

### 3.3 VB-84922 inhibits SREBP-1c-dependent transcription

Under low cholesterol conditions, SREBP-1c transcriptional activity increases the mRNA expression of the sterol-CoA desaturase-1 gene, *SCD1*, (Biddinger et al., 2006). To evaluate the effect of VB-84922 on SREBP-1c transcriptional activity, mRNA and protein levels of *SCD1* were measured in HepG2 cells treated with lovastatin.

**FIGURE 3:**
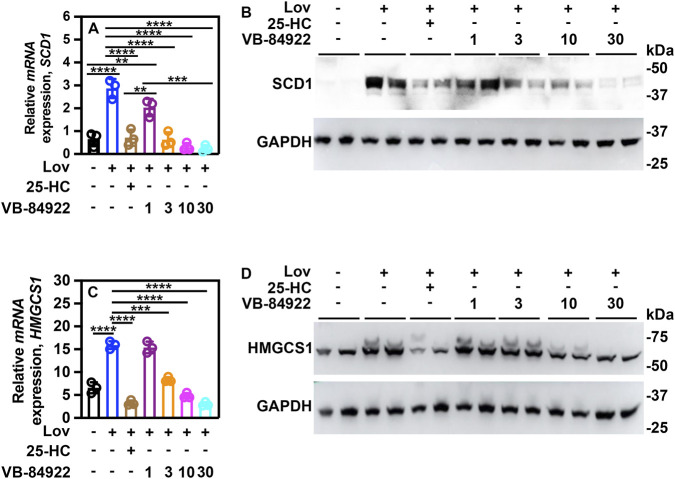
VB-84922 inhibits SREBP-1c-dependent *SCD1* and SREBP-2-dependent *HMGCS1* mRNA and protein expression. HepG2 cells were incubated and treated with the drug as outlined in Figure Legend 6. **(A)** Relative mRNA expression of *SCD1*. **(B)** SCD1 protein levels. **(C)** Relative mRNA expression of *HMGCS1*. **(D)** HMGCS1 protein levels. A two-way ANOVA (Tukey’s *post hoc*) was used for statistical analysis. Data are shown as mean ± SD. **p < 0.001; ***p < 0.0001; ****p < 0.00001.

HepG2 cells treated with 10 μM lovastatin exhibited a threefold increase in *SCD1* gene expression (Figure 3A) as well as elevated protein levels compared to untreated controls (Figure 3B). The addition of 2.5 μM 25-HC reduced *SCD1* gene expression to baseline levels (Figure 3A). Concentrations of VB-84922 above 1 μM similarly decreased *SCD1* gene expression to baseline (Figure 3A). Furthermore, treatment with concentrations of 25-HC or VB-84922 greater than 1 μM resulted in reduced SCD1 protein levels (Figure 3B).

SREBP-1a-dependent gene expression was diminished in cells exposed to the drug. *FASN* expression increased following lovastatin treatment compared to untreated cells (Supplementary Figure S2A) but decreased with the addition of 2.5 μM HC (Supplementary Figure S3A). Similarly, FASN protein levels were reduced in cells treated with 25-HC (Supplementary Figure S3B). Administration of VB-84922 at concentrations of 3, 10, and 30 μM resulted in lower *FASN* expression and FASN protein levels relative to both untreated and lovastatin-treated cells (Supplementary Figures S3A and S3B).

Expression of the *ACC1* gene was suppressed by both 25-HC and all tested concentrations of VB-84922 compared to cells treated with lovastatin (Supplementary Figure S3C). Furthermore, 25-HC as well as VB-84922 at concentrations above 1 μM resulted in a reduction of ACC1 protein levels (Supplementary Figure S2D).

### 3.4 VB-84922 inhibits SREBP-2-dependent transcription

SREBP-2 regulates the expression of *HMGCS1* (HMG-CoA synthase 1) (Horton et al., 2002). In HepG2 cells, lovastatin treatment induced the mRNA expression of *HMGCS1* and HMGCS1 protein levels (Figures 3C,D). The addition of 25-HC decreased *HMGCS1* mRNA expression and HMGCS1 protein levels when compared to untreated and lovastatin-treated cells (Figures 3C,D). Treating cells with 3, 10, or 30 μM VB-84922 reduced *HMGCS1* gene expression when compared to lovastatin-stimulated cells (Figure 3C), with 10 and 30 μM concentrations also decreasing protein levels (Figure 3D).

SREBP-2-dependent expression of the *HMGCR* gene was completely blocked when lovastatin-treated cells were exposed to 25-HC or any concentration of VB-84922 (Supplementary Figure S3E). This effect directly corresponded to a decrease in protein production (Supplementary Figure S3F).

### 3.5 VB-84922 is unable to repress SREBP transcriptional activity in cells expressing a nuclear localized SREBP-1a

Mice exhibiting dominant active SREBP-1a expression show elevated expression of genes involved in cholesterol and fatty acid metabolism, leading to hepatic enlargement (Shimano et al., 1996). If VB-84922 functions by inhibiting SREBPs-SCAP translocation, then overexpression of a soluble, truncated SREBP-1a protein—capable of continuous nuclear translocation—should bypass this inhibition. Accordingly, sustained elevation of *LDLR-SRE*-luciferase activity would be observed. Truncated SREBP-1a was expressed in CHO-K1 Chinese hamster ovary cells, and the effects of VB-84922 on transcription were assessed.

Researchers use CHO-K1 cells as a model to study the biochemical mechanisms that allow SREBPs to move from the endoplasmic reticulum (ER) to the nucleus and to identify the proteins involved in this process (Goldstein et al., 2002; Brown et al., 2018). The findings indicate that this pathway is highly conserved when compared to human cells (Brown et al., 2018).

CHO-K1 cells expressing an *LDLR-SRE*-luciferase plasmid and a dominant active truncated form of SREBP-1a protein (Shimano et al., 1996; Amemiya-Kudo et al., 2002; Harada et al., 2008) were grown under cholesterol deplete conditions (LPDS, containing 210 μM mevalonate plus 2 μM lovastatin) for 16 h in the absence or presence of 1 μg/mL 25-hydroxycholesterol (25-HC), which binds to and inhibits the INSIG-SCAP complex (Adams et al., 2004; Radhakrishnan et al., 2004; Radhakrishnan et al., 2007), or 10 μM VB-84922 and luciferase activity was measured.

**FIGURE 4:**
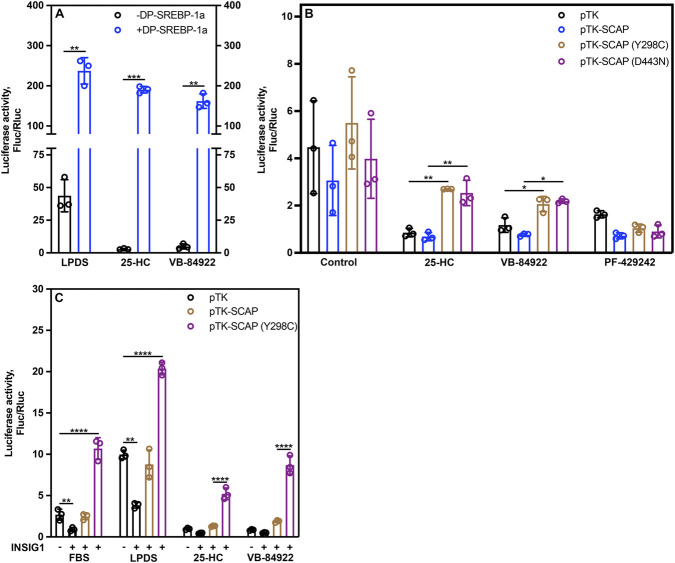
Bypassing SREBP-SCAP ER-Golgi translocation reduces VB-84922’s inhibition of *LDLR-SRE*-luciferase promoter activity. **(A)** On day 0, CHO-K1 cells were set up in a 96-well plate at 15,000 cells in medium A. On day 1, cells were transfected with 125 ng/well of pLDLR-Luc2P, 1 ng/well phRL-CMV with or without 3ng/well of pTK-DP-SREBP1a (“DP-SREBP-1a”). On day 2, cells were washed twice with PBS and switched to cholesterol-depleting medium B plus solvent control, 1 μg/ml 25-hydroxycholesterol (25HC), or 10 μM VB84922 as indicated. Cells were harvested and lysates analysed on day 3. Firefly luciferase activity was divided by Renilla luciferase activity. **(B)** CHO-K1 cells were set up and analysed as above. On day 1, cells were transfected with 125 ng/well of pLDLR-Luc2P, 1 ng/well phRL-CMV plus 10 ng/well of pTK empty vector or SCAP-expressing plasmids as indicated. On day 2, cells were washed and switched to medium B plus solvent, 1 μg/ml 25HC, 10 μM VB84922 or 10 μM PF-429242 as indicated. **(C)** CHO-K1 cells were set up and analysed as above. On day 1 of growth, cells were transfected with 125 ng/well of pLDLR-Luc2P, 1 ng/well phRL-CMV, 20 ng/well pTK-HSV-SREBP2, 0.5 ng/well pCMV-Insig1-Myc plus 10 ng/well of pTK empty vector or SCAP-expressing plasmids as indicated. Error bars denote population standard deviation (n = 3).

Cells expressing the constitutively active SREBP-1a showed a 5.5-fold increase in *LDLR-SRE*-luciferase activity compared to non-expressing cells under cholesterol inducing conditions (Figure 4A (LPDS), -DP-SREBP-1a vs. +DP-SREBP-1a; open black circles vs. open blue circles). Adding either 1 μg/mL 25-HC or 10 μM VB-84922 were ineffective in reducing activity (Figure 4A), suggesting that VB-84922 acts upstream or at the point of SREBP-1a translocation to the Golgi.

### 3.6 VB84922 fails to completely suppress SREBP activity in the presence of constitutively active SCAPY^298C^ or SCAP^D443N^

Cholesterol and 25-HC inhibit SREBPs-SCAP translocation by promoting the interaction between SCAP and INSIGs, stabilizing SCAP in the ER preventing its translocation (Brown et al., 2018; Yan et al., 2021). We asked whether VB-84922 might inhibit SREBP maturation in a manner like sterols by preventing SREBPs-SCAP export from the ER. To test this hypothesis, we took advantage of two well-characterized SCAP variants (Y298C and D443N) that are unable to bind INSIGs in the presence of sterols, leading to constitutive ER export and proteolytic activation of SREBPs (Brown et al., 2018).

When CHO-K1 cells were co-transfected with a plasmid expressing wild-type SCAP (pTK-SCAP), luciferase activity was high in cholesterol-depleting medium (Figure 4B, control, open blue circles) but suppressed ∼4-fold in the presence of 25-HC (Figure 4B, 25-HC, open blue circles) or VB-84922 (Figure 4B, VB-84922), open blue circles). However, incomplete inhibition by 25-HC and VB-84922 was seen in cells co-transfected with SCAP^Y298C^ (Figure 4B, pTK-SCAP (Y298C), open brown circles) or SCAP^D443N^ (Figure 4B, pTK-SCAP (D443N), open purple circles). Activity was consistently low in the presence of the S1P inhibitor PF429242 (Figure 2B, PF-429242). These results further suggest that VB-84922 acts upstream of SREBP export from the ER.

To further explore this hypothesis, we exploited that overexpression of INSIG1 can suppress SCAP transport and SREBP cleavage even in the absence of sterols (Yabe et al., 2002). As shown in Figure 4C, co-transfection of INSIG1 suppressed the SREBP reporter both in the presence of cholesterol-rich FBS (open black circles) as well as in medium containing LPDS and lovastatin (open black circles). When wild-type SCAP was co-transfected with INSIG1, normal expression was restored in LPDS, confirming that wild-type SCAP is expressed and that the stoichiometry of INSIG1 and SCAP was about restored (Figure 4C, pTK-SCAP, open brown circles). VB-84922, like 25-HC (25-HC), suppressed the SREBP reporter ∼5-fold in the presence of both INSIG1 and wild-type SCAP (Figure 4C, open brown circles) but only 2.3-fold in the presence of INSIG1 and SCAP^Y298C^ (Figure 4C, (VB-84922, open purple circles).

Taken together, results in Figure 4C show that SREBP inhibition by VB84922 requires intact residues of SCAP at positions that are also critical for SCAP binding to sterols and INSIG1.

### 3.7 VB-84922 treatment causes SREBP-2 to accumulate in the ER in mouse NMuMG cells

To visually show the effect of VB-84922 on ER-Golgi translocation of SREBP-2, a confocal high-content screening assay (HCS) was performed using mouse epithelial cells (NMuMG) expressing mNeonGreen-SREBP-2. Translocation was induced by treating cells with 10 μM U18666A for 16 h, in the presence or absence of the specified concentrations of the S1P inhibitor PF429242, dipyridamole, or VB-84922.

The utilization of mouse cell lines at this stage is essential to demonstrate that our inhibitor is effective in these cells, suggesting its potential efficacy in mouse models of hyperlipidemia.

**FIGURE 5:**
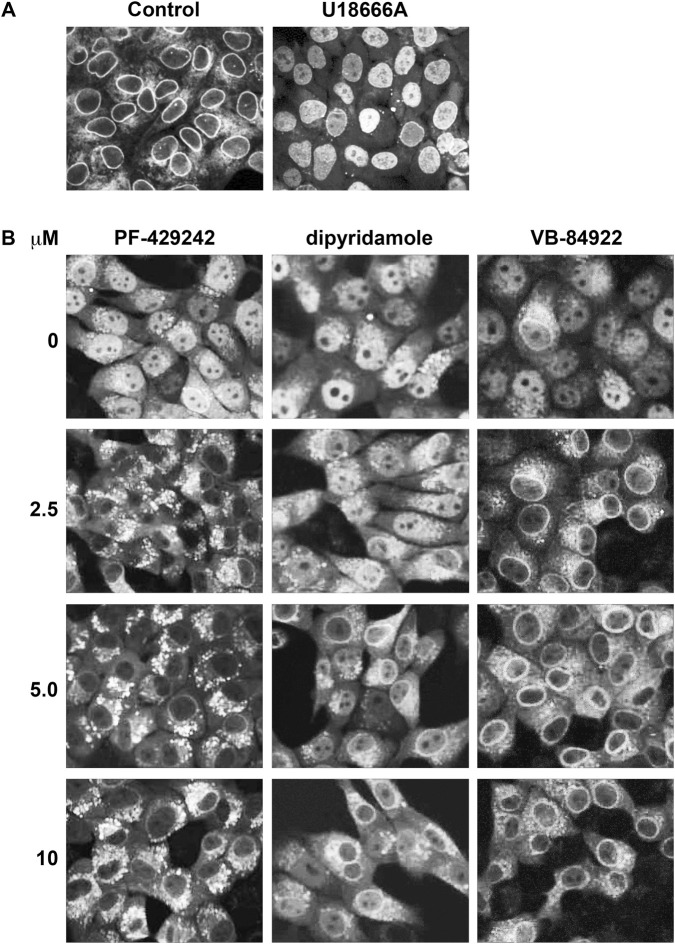
VB-84922 treatment causes SREBP-2 to accumulate within ER-like structures. **(A)** NMuMG cells were grown in the absence and presence of 20 μM U18666A for 16 h. **(B)** Cells grown in the presence of 20 μM U18666A were treated with the indicated concentrations of PF-429242, dipyridamole, or VB-84922. Images were captured using confocal microscopy (OPERA, Phenix, Revvity), sCMOS cameras (4.6 MP, 16-bit), and a ×40 water objective (NA 1.1).

Without activator, SREBP-2 was found in ring structures surrounding a nucleus-like organelle (Figure 5A; control). Activator treatment led to SREBP-2 localization mainly within this organelle in NMuMG cells (Figure 5A; U18666A), suggesting successful ER-to-Golgi transport, proteolytic processing, and nuclear import. Combined activator and PF429242 treatment resulted in SREBP-2 accumulating in punctate structures at all concentrations (Figure 5B). Dipyridamole (≥5.0 μM) led to SREBP-2 presence in dense, cytoplasmic fibers. VB-84922 produced a similar localization pattern across all concentrations, but higher doses caused SREBP-2 to accumulate in perinuclear ring-like structures. A color image is shown in Supplementary Figure S3.

To definitively determine the specific organelles to which SREBP-2 localized under various drug treatments, the proportion of mNeonGreen-SREBP-2 co-localizing with established organelle markers was measured. The organelle markers used were as follows. RanGTP for the nucleus, Ctyb (cytochrome b) for the ER, lamin for the nuclear envelope/ER, and the ERGIC53 (which transits between the ER and Golgi), the ER retention marker CALR-KDEL, and neuromodulin together for the late Golgi/plasma membrane/secretory pathway. This value represented the fraction of SREBP-2 found outside the ER and localized in the Golgi or vesicles associated with the endosomal pathway or retrograde transport to the ER.

**FIGURE 6:**
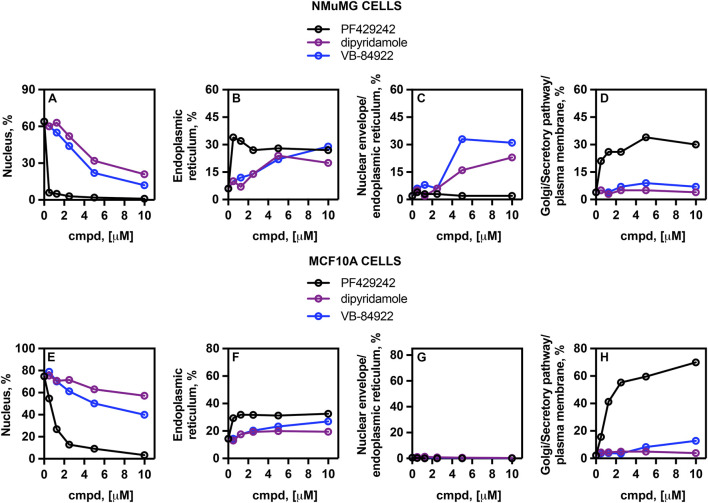
VB-84922 treatment blocks SREBP-2 ER-Golgi translocation. NMuMG cells **(A–D)** or MCF10A cells **(E–H)** expressing mNeonGreen-SREBP-2 were cultured with or without 20 µM U18666A for 16 h and varying concentrations of the VB-84922. The percentages of SREBP-2 localized to specific organelles were quantified as described (Schormann et al., 2020).

In NMuMG cells treated with 10 μM U168666A, approximately 65% of mNeonGreen-SREBP-2 was localized to the nucleus (Figure 6A), while about 6% was observed in the ER (Figure 6B). The addition of 0.25 μM PF429242 reduced SREBP-2 nuclear localization under activating conditions (Figure 6A), with an IC_50_ of 0.002 ± 0.00072 μM. This reduction corresponded with increased localization of SREBP-2 to both the ER (30%) (Figure 6B) and Golgi/secretory pathway/plasma membrane (30%) (Figure 6D).

Treatment with dipyridamole at 10 μM led to a 22% decrease in the percentage of SREBP-2 localizing to the nucleus (Figure 5A), yielding an IC_50_ of 1.8 ± 0.054 μM. This decrease in nuclear accumulation was associated with a 3.3-fold increase in ER localization (Figure 6B). Co-localization with the nuclear envelope/ER marker, lamin, was not detected at 10 μM (Figure 6C). These results were consistent with microscopic observations, which showed that dipyridamole treatment resulted in very few ring-like structures.

Administration of VB-84922 decreased nuclear localization of SREBP-2 by 66% at 10 μM, with an IC_50_ of 1.2 ± 0.0082 μM (Figure 6A), and produced a similar increase in ER localization as seen with dipyridamole (Figure 4B). Unlike dipyridamole, VB-84922 treatment caused SREBP-2 to mainly localize to the nuclear envelope/ER, reaching 30% at 5 μM (Figure 6C).

Neither dipyridamole nor VB-84922, at any tested dose, resulted in significant localization of SREBP-2 to the Golgi/secretory pathway/plasma membrane (Figure 6D).

### 3.8 Human MCF10A cells show sensitivity to VB-84922

MCF10A cells originate from human mammary epithelial cells. To assess the effects of VB-84922 on SREBP-2 translocation in human cells, the HSC assay was used to visualize mNeonGreen-SREBP-2 localization and analyze its organelle distribution.

Demonstrating similar results of VB-84922 in a human cell line was essential to advance our drug discovery efforts, as our primary objective is to develop a therapeutic for humans.

MCF10A cells exhibited a greater tolerance to all three drugs but still demonstrated a measurable response to treatment (Figures 6E–H). When treated with 10 μM U168666A, approximately 75% of SREBP-2 was detected in the nucleus (Figure 6E). PF429242 inhibited nuclear translocation in activated cells, with an IC_50_ (0.5 ± 0.031 μM) that was 250-fold weaker than determined for NMuMG cells (Figure 6E). There was partial accumulation in the ER (14%) (Figure 6F), while most SREBP-2 remained localized to Golgi/secretory pathway/plasma membrane compartments (∼70%) (Figure 6H).

Treatment with dipyridamole and VB-84922 resulted in reduced nuclear SREBP-2 localization, with IC_50_ values of 34 ± 1.3 μM and 5.2 ± 0.51 μM, respectively (Figure 6E). At higher concentrations, SREBP-2 was found to accumulate in the ER (Figure 4F) and Golgi/secretory pathway/plasma membrane compartments (Figure 4H).

### 3.9 VB-84922 blocks SCAP ER-golgi translocation

SREBP-2 translocation is dependent on SCAP (Brown et al., 2018). To further elucidate the mechanism of action underlying VB-84922, we investigated whether SCAP remains within the endoplasmic reticulum (ER) in the presence of VB-84922, or if SREBP-2 translocation is inhibited independently of SCAP. NMuMG cells expressing mNeonGreen-SCAP were treated with 10 μM U18666A, with or without 10 µM lycorine, dipyridamole, or VB-84922. Cellular movement was monitored using the HCS assay.

**FIGURE 7:**
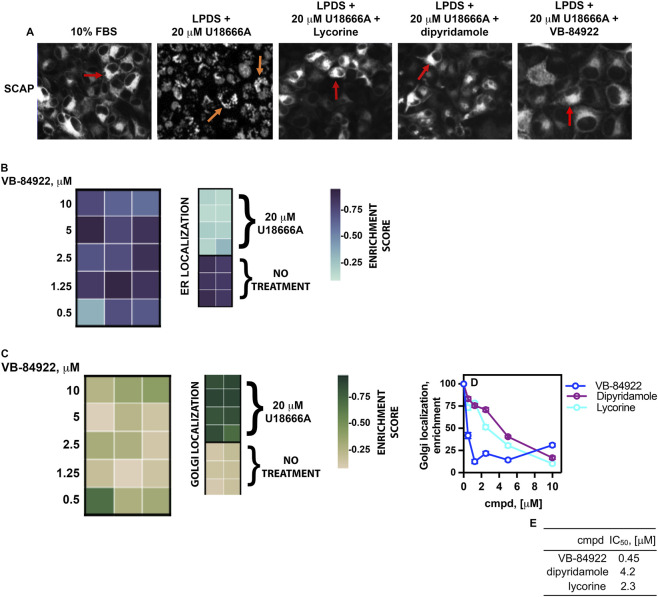
SCAP ER-Golgi translocation is inhibited by VB-84922. **(A)** NMuMG cells expressing mNeonGreen-SCAP were cultured in either 10% FBS or LPDS containing 20 µM U18666A and 10 μM of the indicated drug for 16 h. Images were collected as described in figure legend 3. Golgi, orange arrows; ER, red arrows. **(B)** A heatmap showing the levels of SCAP ER localization at the specified VB-84922 concentrations. **(C)** A heatmap showing the levels of SCAP Golgi localization at the indicated VB-84922 concentrations. **(D)** Graph representing Golgi localization enrichment values plotted against the indicated compound concentrations. **(E)** table indicating IC_50_ values for individual compounds using data from **C**.

SCAP was observed in the diffuse endoplasmic reticulum (ER) in cells under non-inducing conditions (Figure 7A, 10% FBS, red arrow). This correlated with a high ER enrichment score (Figure 7B; no treatment). In cells cultured in LPDS and treated with 20 μM U18666A, SCAP translocated from the ER to the Golgi, indicated by the presence of punctate structures (Figure 7A, LPDS +20 μM U18666A, orange arrow). This correlated with an increase in Golgi enrichment score (Figure 7C, Golgi localization; 20 μM U18666A; Golgi enrichment score). Treatment with lycorine, dipyridamole, and VB-84922 at a concentration of 10 μM inhibited ER-Golgi translocation, resulting in the localization of SCAP within the ER under inducing conditions (Figure 7A, indicated by red arrows). Among these, VB-84922 exhibited the lowest IC_50_ at 0.45 ± 0.033 μM - lycorine and dipyridamole had IC_50_ values of 2.3 ± 0.076 μM and 4.3 ± 0.13 μM, respectively, (Figures 7D,E),- and demonstrated a concentration-dependent rise in ER enrichment score (Figure 7B, ER localization; enrichment score).

### 3.10 VB-87496, a chemically optimized derivative of VB-84922, demonstrates *in vivo* efficacy

Medicinal chemistry initiatives focused on enhancing the pharmacokinetic profile and potency of VB-84922 led to the development of VB-87496.

**FIGURE 8:**
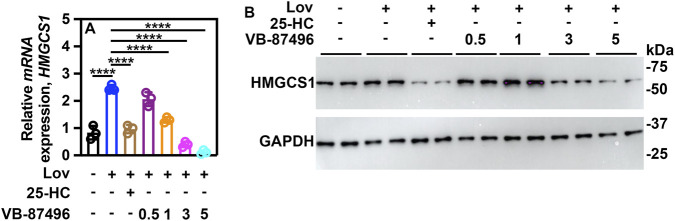
VB-87496 blocks lovastatin-induced *HMGCS1* expression and protein levels. HepG2 cells were incubated with or without 10 μM lovastatin for 16 h and the indicated concentrations of VB-87496. **(A)** relative mRNA expression of *HMGCS1*. **(B)** protein levels of HMGCS1. A two-way ANOVA (Tukey’s *post hoc*) was used for statistical analysis. Data are shown as mean ± SD. ****p < 0.00001.

In HepG2 cells, VB-87496 reduced *HMGCS1* expression with an IC_50_ value of 0.94 ± 0.077 μM (Figure 8A). Furthermore, VB-87496 induced a dose-dependent reduction in HMGCS1 protein levels (Figure 8B).

Given its favorable biological and chemical properties, we proceeded to assess the *in vivo* efficacy of VB-87496 using an acute fasting/refeeding mouse model (Esquejo et al., 2021).

**FIGURE 9:**
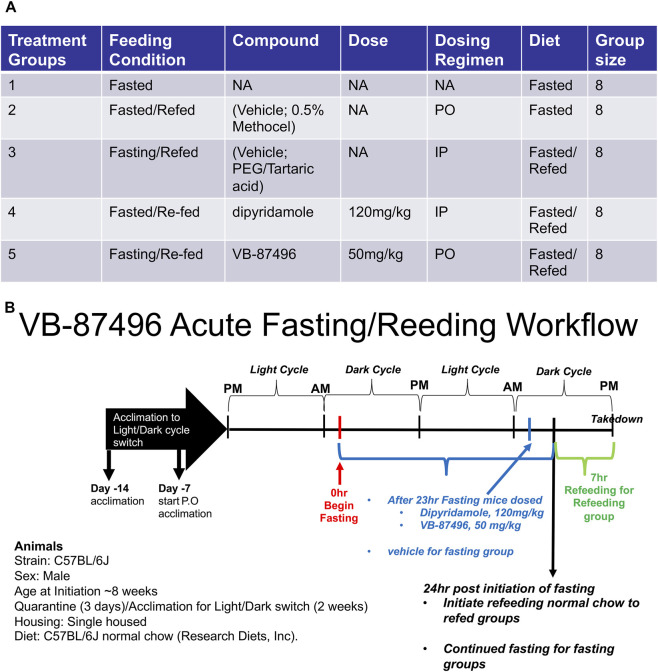
VB-87496 *in vivo* study plan. **(A)** treatment conditions for individual treatment groups. **(B)** Workflow for fasting-refeeding study. Mice underwent a 24-h fasting period. One hour before refeeding began, selected groups were given compounds at specified concentrations. After the 24-h fast, the mice were refed for 7 h, then euthanized so their livers could be collected.

The cohort groups are detailed in Figure 9A. Separate vehicle control groups were necessary (Groups 2 & 3) since dipyridamole was formulated in PEG/tartaric acid (120 mg/kg, IP; Group 4) and VB-87496 in 0.5% Methocel (50 mg/kg, PO; Group 5). Furthermore, the light/dark cycle was adjusted so that refeeding occurred during the dark phase, aligning with the period when mice normally consume food.

Figure 9B illustrates the fasting/refeeding workflow. Mice underwent a 24-h fast, and those assigned to refeeding were also given a compound 1 hour before being fed again. After 7 h of feeding, the mice were euthanized, and their livers were collected for total RNA and protein extraction.

**FIGURE 10:**
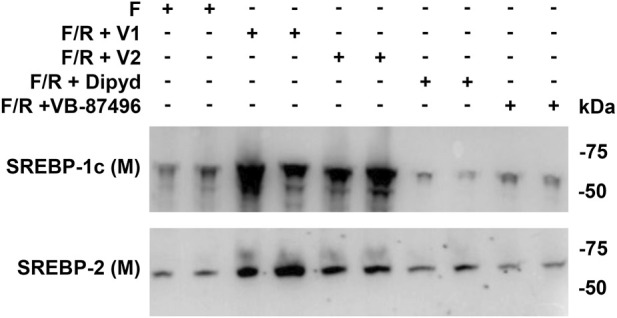
VB-87496 blocks SREBP-1c and SREBP-2 proteolytic processing and maturation *in vivo*. Mice were treated as shown in Figure 9. Liver protein levels of mature SREBP-1c and SREBP-2 were measured by western blot.

Mice fasted for 24 h had low SREBP-1c and SREBP-2 protein levels (Figure 10). Refeeding increased both isoforms, but this effect was blocked by VB-87496 and dipyridamole, returning levels to those seen during fasting (Figure 10).

**FIGURE 11:**
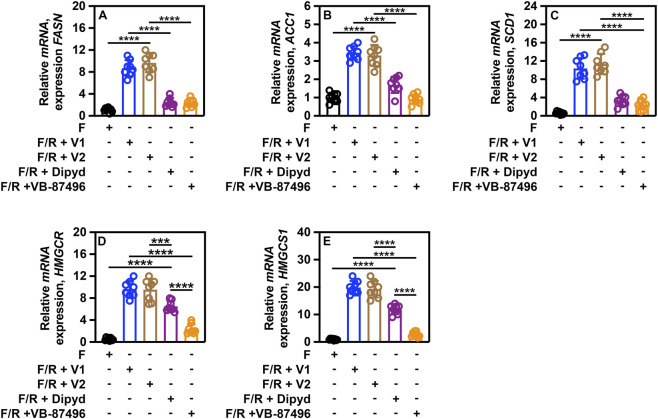
VB-87496 reduces SREBP-dependent gene expression *in vivo*. mRNA levels were determined by *q*RT-PCR using *GAPDH* expression as a loading control. **(A)** relative *FASN* expression. **(B)** relative *ACC1* expression. **(C)** relative *SCD1* expression. **(D)** relative *HMGCR* expression. **(E)** relative *HMGCS1* expression. A two-way ANOVA (Tukey’s *post hoc*) was used for statistical analysis. Data are shown as mean ± SD. ***p < 0.0001; ****p < 0.00001.

There was a direct correlation between the levels of SREBP isoforms and SREBP-dependent gene expression. Mice subjected to a 24-h fast exhibited markedly reduced expression of SREBP-1a (*FASN*/*ACC1*), SREBP-1c (*SCD1*), and SREBP-2 (*HMGCR*/*HMGCS1*)-dependent genes (Figures 11A–F). Refeeding induced the expression of all these genes in both vehicle-treated cohorts (Figures 11A–E, F/R + V1 and F/R + V2). Mice administered VB-87496 maintained gene expression levels comparable to those observed during fasting (Figures 11A–E, F/R + VB-87496 vs. F). Administration of dipyridamole was similarly effective in reducing SREBP-1a (*FASN*/*ACC1*) and SREBP-1c (*SCD1*) gene expression; however, its efficacy in lowering SREBP-2-dependent gene expression was diminished compared to VB-87496 (Figures 11D and E, F/R + dipyd vs. F/R + VB-87496).

## 4 Discussion

The SREBP transcription factors regulate fatty acid and cholesterol biosynthesis (Brown and Goldstein, 1997), making them attractive targets for treating diseases associated with elevated lipid levels. The use of statins to inhibit HMG-CoA reductase has been effective in treating these disorders; however, statin therapy induces SREBP-dependent fatty acid and *de novo* cholesterol gene expression, which may reduce overall efficacy (Cai et al., 2021). SREBPs-SCAP complex translocation is essential for SREBPs processing and translocation to the nucleus for subsequent transcription (Nohturfft et al., 1998b; Matsuda et al., 2001; Brown et al., 2018). Blocking ER-Golgi translocation directly blocks gene expression at a stage prior to initiating lipid synthesis. Compounds inhibiting translocation show efficacy across various murine models, resulting in decreased levels of hyperlipidemia and reduced atherosclerosis (Kamisuki et al., 2009; Tang et al., 2011; Esquejo et al., 2021; Zheng et al., 2021). Thus, the pursuit of small molecules that effectively inhibit translocation continues and is warranted (Wang et al., 2025).

Increased *SREBF1* and *SREBF2* expression is observed in individuals with metabolic disorders (Moon, 2017; Li N. et al., 2023) and cancer (Rohrig and Schulze, 2016; Li Y. et al., 2023). Reducing SREBP activity in mice is associated with improvements in these conditions, indicating the potential benefits of targeting it for therapeutic drug design (Zhao et al., 2022; Peng et al., 2023). Studies do exist however suggesting decreasing SCAP activity may have adverse outcomes (Verheijen et al., 2009; Suzuki et al., 2013; Li et al., 2021; Kawamura et al., 2022; Osborne and Espenshade, 2022; Lee et al., 2023). Mice lacking SCAP in Schwann cells display reduced myelin formation (Verheijen et al., 2009), and loss of SCAP in the brain has been linked to impaired synaptic function and cognitive deficits (Suzuki et al., 2013). In addition, loss of SCAP in the livers of mice has been shown to exacerbate liver injury, fibrosis, and cancer progression (Kawamura et al., 2022), and its loss in macrophages results in obesity and insulin resistance (Lee et al., 2023).

It should be noted, however, that ∼98% of small molecules are unable to cross the blood brain barrier (Pardridge, 2005) and those capable are often subject to modification through medicinal chemistry to manage such concerns (Pardridge, 2005; Whelan et al., 2021). Moreover, the mice used in the above mentioned studies were ablated for SCAP. Small molecule inhibitors likely would not eliminate but partially inhibit SCAP activity (Csermely et al., 2005; Masson and Mukhametgalieva, 2023), and clinical dose-range finding studies for determining the minimal effective dose (MED) can help determine a safe, effective dose (Boissel et al., 1995; Zhou et al., 2017). Overall, the field of SREBP/SCAP inhibitor discovery has seen significant activity (Peng et al., 2023).

Our findings strongly suggest VB-84922 functions by inhibiting SREBPs-SCAP translocation from the ER to the Golgi. Treatment of U2OS cells with VB-84922 resulted in decreased SREBP-2 nuclear localization in our HTS screening assay. Furthermore, VB-84922 did not diminish *LDLR*-*SRE* luciferase activity in CHO-K1 cells expressing either a constitutively active SREBP-1a or SCAP mutants resistant to cholesterol regulation. VB-84922 treatment resulted in both SREBP-2 and SCAP remaining in the ER of mouse and human mammary cells. It also blocked SREBP-1c and SREBP-2 processing, indicating a block at the point of ER to Golgi transport for S1P and S2P protease cleavage. Our preliminary data suggests that inhibition of the translocation of the SREBPs-SCAP complex is specific, as VB-84922 does not inhibit the secretory transport of Vesicular Stomatitis Virus (Nohturfft, preliminary results).

We have demonstrated that the medicinal chemistry-optimized derivative, VB-87496, exhibits substantial *in vivo* efficacy. In fasted/refed mice, treatment with this compound results in the inhibition of SREBP-1c and SREBP-2 protein maturation observed in untreated feeding conditions, indicating a disruption in ER-Golgi transport. This disruption leads to the elimination of feeding-dependent, SREBP-mediated gene expression. The observed *in vivo* efficacy provides robust validation for the findings derived from multiple cell lines used to elucidate the mechanism of action.

What is the mechanism(s) by which VB-84922 and VB-87496 inhibit SREBPs-SCAP translocation? Cholesterol and 25-hydroxycholesterol (25-HC) have been shown to inhibit SREBPs-SCAP translocation, but they interact with the INSIG-SCAP complex in distinct ways (Brown et al., 2018). 25-HC binds to INSIG2 (Radhakrishnan et al., 2007) within a hydrophobic pocket formed by the INSIG2-SCAP complex (Yan et al., 2021), whereas cholesterol binds to the sterol-sensing domain (SSD) of SCAP (Adams et al., 2004; Radhakrishnan et al., 2004). Although it is possible that the chemical structure of our compounds enables binding in a manner comparable to cholesterol or 25-HC, they do not share structural similarity with any known sterol.

Dipyridamole, a pyrimidopyrimidine derivative and phosphodiesterase inhibitor, is used in the management of blood clots (Kim and Liao, 2008). Recent findings indicate that a photoactivatable dipyridamole derivative lacking phosphodiesterase activity can bind SCAP and/or INSIG within cells (Esquejo et al., 2021). Notably, dipyridamole inhibits SREBP-2 translocation in our HTS and HSC assays—a property not observed with other SCAP binding compounds. Given that the structure of dipyridamole differs significantly from sterols, it may function as a molecular glue, stabilizing the INSIG-SCAP complex (Dewey et al., 2023). Our compounds may exhibit a similar mechanism of action. It is also conceivable that they interact with a previously uncharacterized site within any of the three INSIG-SCAP-SREBP proteins. Very recently the ER-phagy receptor FAM134B was shown to bind SCAP and inhibit SCAP-SREBP-2 ER-Golgi translocation (Li et al., 2025). It may also be possible that VB-84922 and VB-87496 bind this receptor and enhance its binding to SCAP.

## 5 Conclusion

SCAP binding inhibitors have shown effectiveness in pre-clinical metabolic studies. Fatostatin reduces some cancers, and dipyridamole with fluvastatin inhibits cancer cell growth (Pandyra et al., 2014; Longo et al., 2019; Longo et al., 2022), indicating further potential for these molecules. VB-87496 demonstrated *in vivo* efficacy by blocking SREBP processing and transcriptional activity. Biochemical analysis is now required to assess whether VB-84922 and VB-87496 directly interact with SREBPs, SCAP, or INSIG, clarifying if their inhibition of SREBP-SCAP translocation is due to direct physical interaction.

## Data Availability

The raw data supporting the conclusions of this article will be made available by the authors, without undue reservation.
