# Supplementary material for: VB-84922 is a small molecule that inhibits ER-to-golgi transport of SREBPs-SCAP complexes
Source: Front Pharmacol. 2026 Mar 24;17:1732319. doi: 10.3389/fphar.2026.1732319 (PMC13055617; doi:10.3389/fphar.2026.1732319)
Supplement: Supplementary file 1 [file Table1.docx]

Table S1. Antibodies used for western analysis

| Protein | Company | Catalog# | Dilution |
| --- | --- | --- | --- |
| SREBP1c | Affinity BioSciences | BF8311 | 1:750 |
| SREBP2 | Abcam | ab30682 | 1:750 |
| HMGCS1 | Abcam | ab155787 | 1:1,000 |
| ACC1 | Abcam | ab109368 | 1:1,000 |
| FASN | Abcam | ab128870 | 1:1,000 |
| HMGCR | Abcam | ab242315 | 1:2,500 |
| GAPDH | Millipore Sigma | 2118 | 1:1,000 |
|  |  |  |  |
|  |  |  |  |
|  |  |  |  |
|  |  |  |  |
|  |  |  |  |
|  |  |  |  |
|  |  |  |  |
|  |  |  |  |
|  |  |  |  |
|  |  |  |  |
