# Supplementary figures and images for: VB-84922 is a small molecule that inhibits ER-to-golgi transport of SREBPs-SCAP complexes
Source: Front Pharmacol. 2026 Mar 24;17:1732319. doi: 10.3389/fphar.2026.1732319 (PMC13055617; doi:10.3389/fphar.2026.1732319)

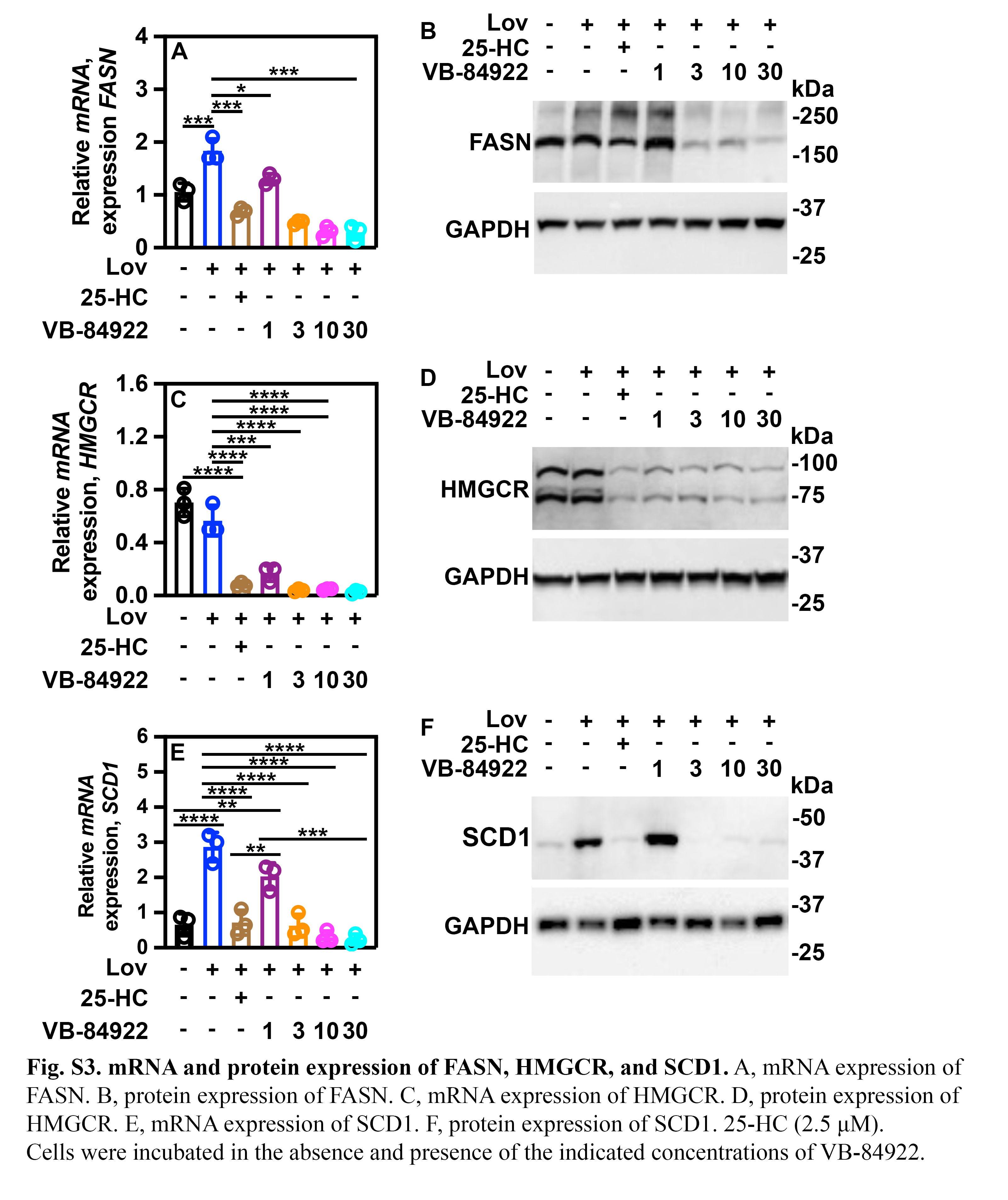

Supplement: Supplementary file 2 [file Image3.tif]

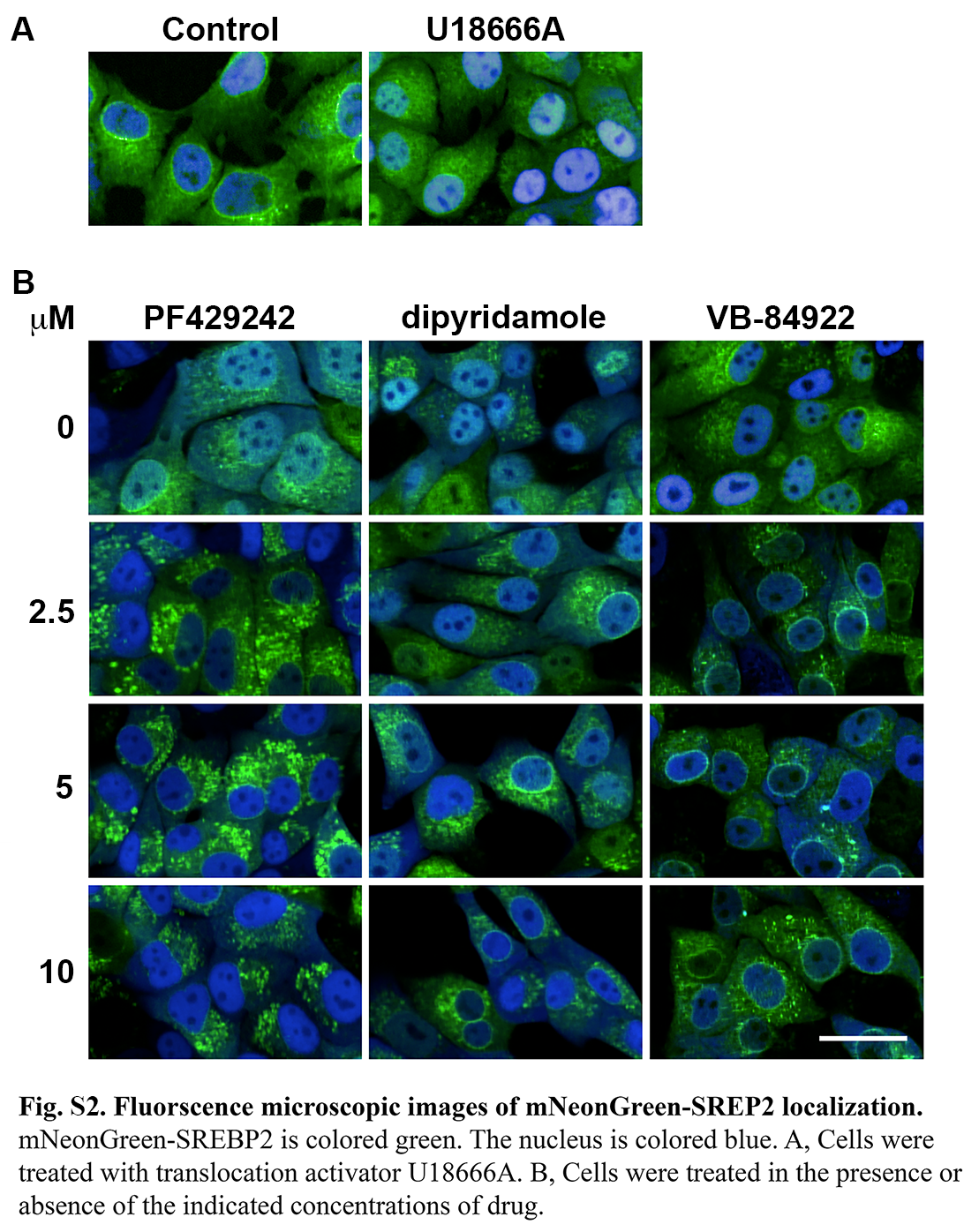

Supplement: Supplementary file 3 [file Image2.tif]
