# Supplementary material for: VB-84922 is a small molecule that inhibits ER-to-golgi transport of SREBPs-SCAP complexes
Source: Front Pharmacol. 2026 Mar 24;17:1732319. doi: 10.3389/fphar.2026.1732319 (PMC13055617; doi:10.3389/fphar.2026.1732319)
Supplement: Supplementary file 4 [file Image1.pdf]

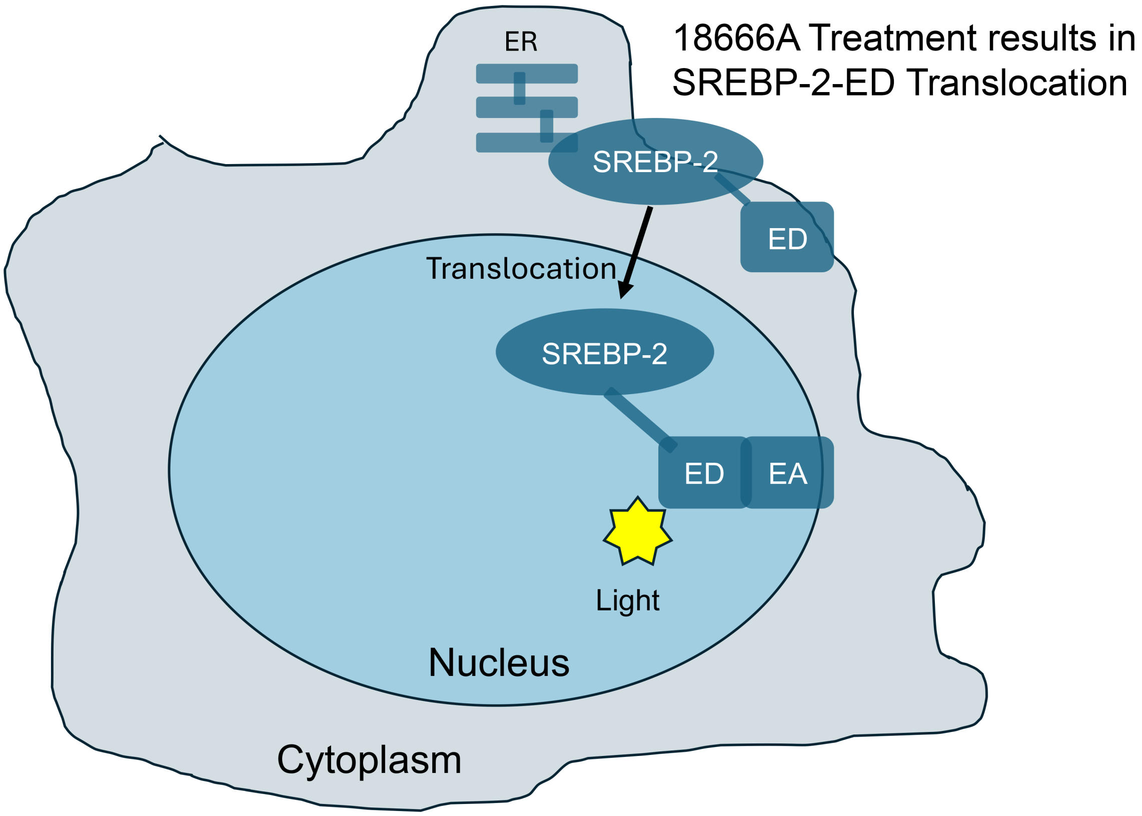

Figure S1. Pathhunter Xpress SREBP2 translocation assay. The assay is based on enzyme complementation. ER-tethered SREBP-2 fragment is tagged with a donor fragment (ED). An EA acceptor fragment is tehered to the nuclear membrane for complementation.Treatment with U1866A, which blocks cholesterol transport causes the translocation of the SREBP-2-ED to the nucleus. The association between the EA and ED frament generates a light signal that can be detected.
